# Only the chemical state of Indium changes in Mn-doped In_3_Sb_1_Te_2_ (Mn: 10 at.%) during multi-level resistance changes

**DOI:** 10.1038/srep04702

**Published:** 2014-04-16

**Authors:** Y. M. Lee, D. Ahn, J.-Y. Kim, Y. S. Kim, S. Cho, M. Ahn, M.-H. Cho, M. S. Jung, D. K. Choi, M.-C. Jung, Y. B. Qi

**Affiliations:** 1 Pohang Accelerator Laboratory, POSTECH, Pohang, 790-784, Republic of Korea; 2 Energy Harvest-Storage Research Center (EHSRC) and Department of Physics, University of Ulsan, Ulsan 680-749, Republic of Korea; 3 Department of Physics, Yonsei University, Seoul 120-749, Republic of Korea; 4 Department of Material Science and Engineering, Hanyang University, Seoul 133-791, Republic of Korea; 5 Energy Materials and Surface Sciences Unit, Okinawa Institute of Science and Technology Graduate University, Okinawa, 904-0495, Japan

## Abstract

We fabricated and characterized the material with Mn (10 at.%: atomic percent) doped In_3_Sb_1_Te_2_ (MIST) using co-sputtering and synchrotron radiation, respectively. The MIST thin film showed phase-changes at 97 and 320°C, with sheet resistances of ~10 kΩ_sq_ (amorphous), ~0.2 kΩ_sq_ (first phase-change), and ~10 Ω_sq_ (second phase-change). MIST did not exhibit any chemical separation or increased structural instability during either phase-change, as determined with high-resolution x-ray photoelectron spectroscopy. Chemical state changes were only depended for In without concomitant changes of Sb and Te. Apparently, doped Mn atoms can be induced with movement of only In atoms.

Recently, phase-change materials have become highly promising for use in rewritable optical media and non-volatile random access memory devices (PRAM)^1^,^2^,^3^,^4^,^5^. These materials manifest extreme changes in both optical reflectivity and electrical resistance during the amorphous-to-crystalline phase-change at 100 ~ 400°C^4^,^5^. Ge_2_Sb_2_Te_5_ ternary alloy (GST) is widely used in both applications because of its proper phase-change temperature of 180°C and its high phase-change speed^4^,^5^. Samsung Electronics Co. Ltd. (SEC) recently announced a GST-based 8 Gb PRAM device with 20 nm process technology and an operational power requirement of 1.8 V^6^. GST has enabled next generation memory devices to be developed more rapidly than other technologies, such as ferroelectric and resistive random access memory devices.

Many studies have reported improvements in suitable packaging for phase-change materials, high integration scale, and more stable phase-change processes with variable stoichiometry, using chalcogenide-based elements^7^,^8^,^9^. Recently, In_3_Sb_1_Te_2_ (IST) phase-change material with multi-phase-change properties has been employed in multi-bit memory applications because of its high speed, low voltage, stable reversibility, and large integration^10^,^11^. IST was shown to transition from amorphous IST (*a*-IST), InSb, InTe to crystalline IST (*c*-IST) at different phase transition temperatures^10^,^11^. It demonstrated great promise for use in multi-level optical media and multi-bit memory devices. However, in spite of its multi-phase-change properties, it has a serious problem: multi-phase-changes result in chemical separation^12^. Our previous study found that it has a high probability of suffering Sb atomic degradation and chemical separation during phase-transitions, so that it has a high failure potential in memory performance^12^. This degradation due to chemical separation is critically important because it determines the lifetime and reliability of devices that employ this material. In this work, we doped IST to improve atomic and chemical stability without losing its multiple-phase-changes.

In this study, atomic and electrical structures of Mn-doped In_3_Sb_1_Te_2_ (Mn: 10 at.%) thin film (MIST) were determined using high-resolution x-ray diffraction (HRXRD), near-edge x-ray absorption of fine structure (NEXAFS), and high resolution x-ray photoelectron spectroscopy (HRXPS) with synchrotron radiation. We investigated structural stability in three chemical states of MIST and found that only In atoms move during phase-changes.

## Results

**Figure 1:**
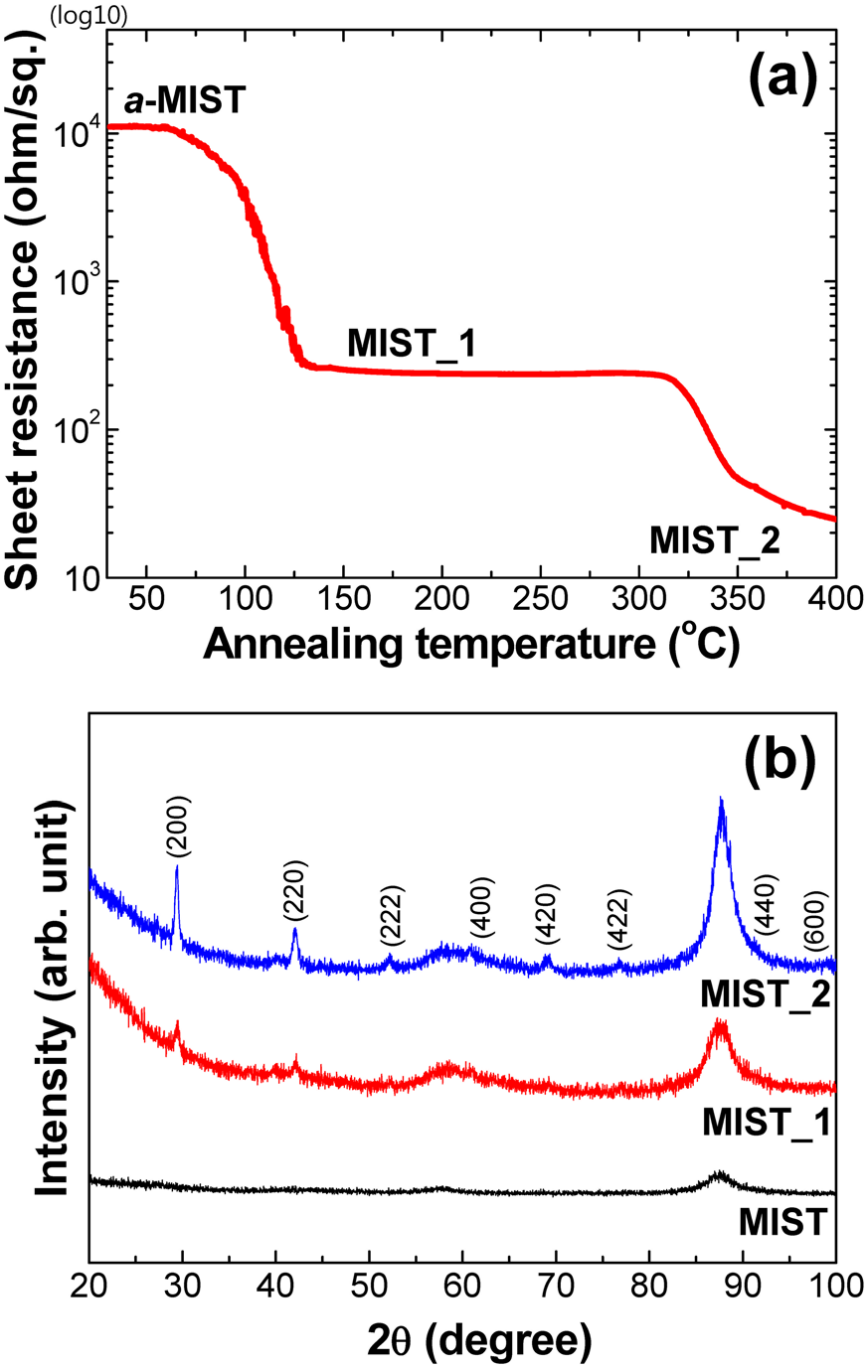
(a) Sheet resistance as a function of increasing temperature. The phase-change temperatures are 97° and 320°C, respectively. Resistance changed from 10 kΩ_sq_ to 10 Ω_sq_. (b) XRD patterns of *a*-MIST, MIST_1 and MIST_2 samples. MIST_1 was shown to have a semi/quasi-crystalline phase.

Significant changes in sheet resistance correspond to two phase transitions in MIST (Figure 1a). Specifically, sheet resistance dropped from ~10 kΩ_sq_ for *a*-MIST to ~0.2 kΩ_sq_ for MIST_1, the phase formed at 97°C. A further reduction in resistance of almost three orders of magnitude (to ~10 Ω_sq_) occurred at 320°C, which corresponds to the final transition state of MIST, called MIST_2. Resistance remained constant from 125 to 325°C, a temperature gap of 200°C, between the two transitions (Figure 1a). This means that MIST_1 is stable.

In order to understand the variation in structural characteristics of MIST, HRXRD patterns of the three different phases of MIST were recorded (Figure 1b). Low intensity peaks in the XRD pattern of MIST_1 roughly correspond to the diffraction planes of (200) and (220), and the system can be modeled to a cubic phase belonging to the Fm-3m (225) space group^13^. In fact, the above mentioned diffraction peaks are not distinguishable in the parent *a*-MIST pattern, which clearly confirms the occurrence of a transition from an amorphous-like to a quasi-crystalline phase (MIST_1). It is highly possible that the quasi-crystalline phase of MIST_1 may not possess a complete 3D atomic structural arrangement with long-range lattice ordering, due to the low annealing temperature involved and the effect of substrate orientation^14^,^15^,^16^. Weak diffraction planes in the pattern of MIST_1 suggest preferred crystallographic growth orientations along the (200) and (220) Bragg planes^14^,^15^,^16^. In fact, an amorphous to quasi-crystalline phase transition above 97°C is also confirmed by the observed reduction in sheet resistance (Figure 1a). However, the XRD pattern of MIST_2 (Figure 1b) indicates the presence of several diffraction peaks, all of which are indexed to the same cubic crystal system^13^,^14^,^15^,^16^. It means that the quasi-crystalline phase is disappeared by high temperature because the thin film has enough energy at high temperature to be its own stable atomic structure without any influence of substrate. Strong diffraction peaks clearly indicate increased crystallinity, which probably accounts for the dramatic lowering of sheet resistance by almost three orders of magnitude in MIST_2 above 320°C (Fig. 1a).

**Figure 2:**
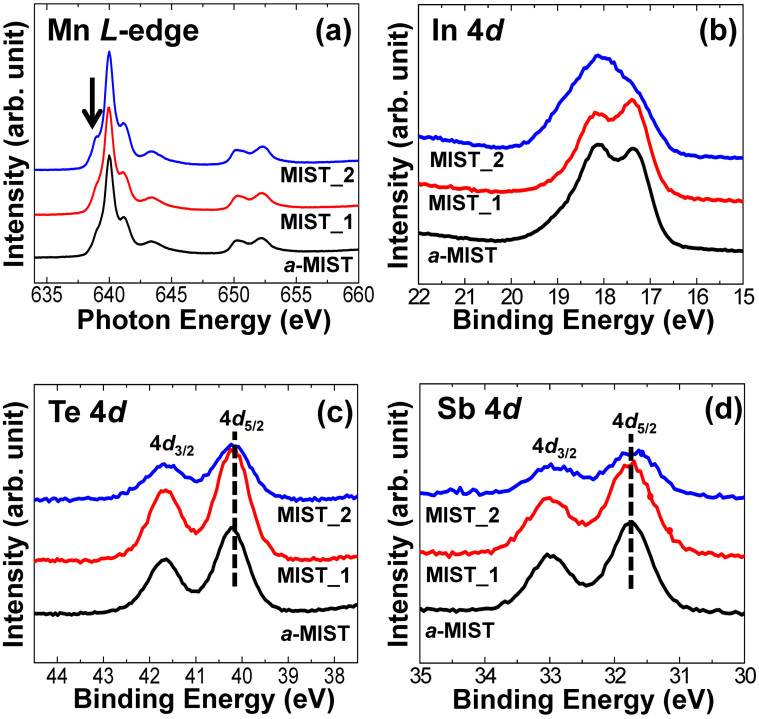
(a) Mn *L*-edge absorption spectra, (b) In 4*d*, (c) Te 4*d* and (d) Sb 4*d* core-level spectra of *a*-MIST, MIST_1 and MIST_2 samples. A shoulder was enhanced at 639 eV photon energy (at the arrow) in Mn *L*-edge spectrum of MIST_2. Binding energies of Te and Sb 4*d*_5/2_ are 40.2 and 31.7 eV, respectively. These peaks are not changed during phase-transitions.

We obtained Mn *L*-edge absorption spectra for *a*-MIST, MIST_1, and MIST_2 (Figure 2a). No significant changes in chemical states were observed when comparing spectra of *a*-MIST and MIST_1, indicating that manganese is not involved in the first phase change. The spectra are consistent with Mn atoms were located in the center of a Mn^2+^ tetrahedral site (T_d_)^17^. However, in the MIST_2 sample, the shoulder at 639 eV of photon energy was enhanced such a change^17^. In HRXPS results, on the other hand, we could not find any significant changes in Te and Sb 4*d* core-level spectra in any sample (Figure 2c,d). The 4*d*_5/2_ binding energies of 40.2 eV and 31.7 eV in Te and Sb, respectively, did not change during phase-transitions. However, an intensity decrease of the Te and Sb 4*d* peaks was observed. This indicated a surface depletion (vaporization) of these atoms resulting from the high phase-change temperature (320°C) and the ultrahigh vacuum in the absence of a capping layer. Only In 4*d* core-level spectra underwent dramatic changes in these samples (Figure 2b). In changes of both Mn *L*-edge and In 4*d* core-level spectra, we can assume that atoms surrounding the octahedral Mn are In.

## Discussion

**Figure 3:**
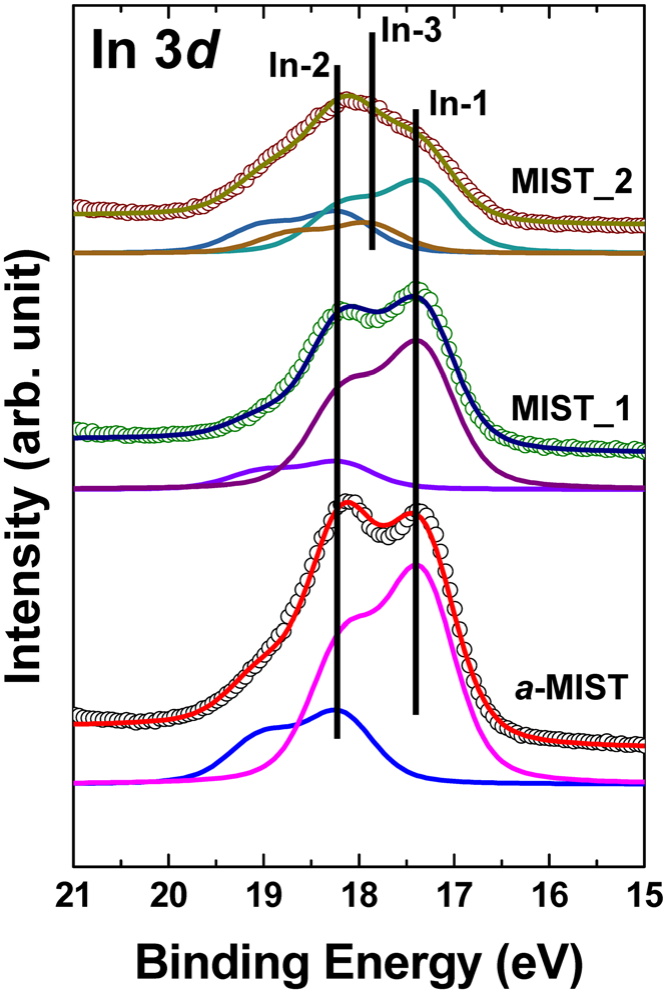
Curve fitting of In 4*d* core-level spectra for *a*-MIST, MIST_1, and MIST_2. Binding energies of In-1, In-2 and In-3 chemical states are 17.4, 18.3 and 17.9 eV, respectively. The chemical state, In-3, appeared only in MIST_2.

To achieve greater detail, we fitted In 4*d* core-level spectra using Doniach-Sŭnjić curves convoluted with a Gaussian distribution of 0.5 eV full-width at half maximum^18^. Background due to inelastic scattering was subtracted by the Shirley (integral) method^19^. Three chemical states of In, In-1, In-2, and In-3 with binding energies of 17.4, 18.3 and 17.9 eV, respectively, were observed in each sample (Figure 3). Basically, *a*-MIST seemed to have two chemical states, In-1 and In-2, that originated from In-Sb-Te and Mn-(In-Sb-Te), respectively. After the first phase-change, the intensity of In-2 decreased, relatively increasing the peak intensity of In-1. This means that the mechanism of the first phase-change originated from the quasi-crystalline stage of In-Sb-Te (MIST_1) without involvement of the core Mn atom. However, in MIST_2, In-3 appeared at a binding energy of 17.9 eV, between the In-1 and In-2 states. Binding energies of In-2 and In-1 were fixed while their peak intensities decreased. We can assume that the origin of the second phase-change was the new chemical state of In (Figure 3). During the first phase-change, Mn atoms do not directly affect the resistance change because In-Sb-Te showed only a quasi-crystalline state and MIST_1 has no such chemical state, as in InTe or InSb. After the second phase-change, however, two In atoms from the chemical state, *c*-In-Sb-Te, moved to the Mn atom within the tetrahedral structure. Then the Mn atom was surrounded with six In atoms, with an octahedral structure. This means that the doped Mn atom in IST could be induced by the movement of only In atoms, without any movement of Sb and Te atoms. The multi-phase-change property remained unaltered.

In summary, we fabricated and characterized Mn-doped IST (Mn: 10at.%) using co-sputtering with HRXRD, NEXAFS, and HRXPS, respectively. This MIST thin film showed electrical-phase-changes at 97 and 320°C. The key to the phase-changes was the quasi-crystalline phase during In-Sb-Te crystallization without the incorporated Mn atom in the first phase-change. The main mechanism in the second phase-change seemed to be the structural change of the Mn atom from a tetrahedral to an octahedral structure, surrounded by In atoms. Also MIST did not manifest any chemical separation during these phase-changes. It seems that the doped Mn atom can be induced with the movement of only In atoms and without any movement of Sb or Te atoms.

## Methods

### Sample preparation

Mn-doped In_3_Sb_1_Te_2_ (MIST) was formed by co-sputtering Mn and In_3_Sb_1_Te_2_ (IST) targets onto Si substrates. Deposition rates of Mn and IST were 0.8 and 9.5 Å/sec, respectively, with 500 W deposition power and 10 m*Torr* pressure for 120 s. The thickness of the formed thin film was 100 nm.

### The measurements with synchrotron radiation

Before we performed HRXPS and NEXAFS experiments, the MIST thin film was fabricated by mild Ne^+^-ion sputtering in an ultra-high vacuum chamber for 1 hr at a beam energy of 1 kV to remove native surface oxide^20^. We confirmed an oxygen-free atmosphere and a 3:1:2 stoichiometry of the MIST thin film without the O 1*s* core-level peak and with calibration of the intensity peak area, respectively. In order to confirm structural phases of samples, we performed HRXRD at the 9B HRPD beamline of Pohang Accelerator Laboratory (PLS). Incident x-rays in the HRXRD beamline were vertically collimated with a mirror, and monochromatized to a wavelength of 1.5490 Å using a double-crystal Si(111) monochromator. Mn *L*-edge absorption and core-level spectra were obtained with NEXAFS and HRXPS, respectively, at 2A MS and 8A1 SPEM beamlines of PLS. We used a total electron yield for NEXAFS spectra by recording the sample current normalized to a signal current measured simultaneously using a gold mesh in ultra-high vacuum. In the HRXPS experiment, A PHI 3057 with an Omega lens and a 16-channel detector (Physical Electronic Co.) was used as the electron analyzer. The energy resolution of HRXPS was better than 200 meV. The photon energy was 250 eV. Core-level spectra of In, Sb, and Te 4*d* were also obtained. Binding energies were calibrated with respect to the Au 4*f*_7/2_ core-level (84.0 eV)^21^.

## Author Contributions

M.-C.J. and D.K.C. designed and supervised the project. Y.M.L. performed a major portion of sample preparation, optimization, and HRXPS measurements. D.A. and J.-Y.K. performed HRXRD and NEXAFS measurements, respectively. Y.S.K., S.C., M.A., M.-H.C. and M.S.J. participated in the sample design, preparation and basic measurements (Temp.-Resist.). All authors discussed the results. Y.M.L. wrote the manuscript and M.-C.J., D.K.C. and Y.B.Q. revised it.
